# Black Family Caregiving Experiences Among Relatives of Persons with Dementia

**DOI:** 10.1002/alz.086279

**Published:** 2025-01-09

**Authors:** Sheria G Robinson‐Lane, Florence U Johnson, Marie Jean Tuyisenge, Jada A Jackson, Tasneem Qurashi, Priya Tripathi

**Affiliations:** ^1^ University of Michigan, Ann Arbor, MI USA

## Abstract

**Background:**

Despite an increasing prevalence of Alzheimer’s disease and related dementias among Black older adults, large disparities remain in early diagnosis, treatment, and support service referral. Further, services intended to support family caregivers are typically designed around older adult spousal caregivers with little consideration to the wide diversity of families affected by dementia. This qualitative descriptive study examined the caregiving experiences of Black relatives of persons with dementia with the aim of informing the development of more culturally responsive support programming.

**Methods:**

Three focus groups of family caregivers (n1 = 6, n2 = 6, n3 = 5) who self‐identified as Black/ African American and reported providing supportive care for a relative over the age of 55 with dementia or evidence of cognitive impairment were convened to answer the primary research question, “what characterizes the experience of Black adults providing supportive care to a relative with dementia?” Virtual focus groups took place via Zoom and transcripts were analyzed thematically by a team of 5 trained coders following the rigorous and accelerated data reduction technique (RADaR).

**Results:**

Participants, primarily non‐spousal caregivers, identified seven barriers and three facilitators of family caregiving. Barriers included 1) financial challenges, 2) familial expectations and lack of support, 3) managing difficult emotions, 4) managing challenging behaviors of care recipients, 5) dealing with gaps in medical care/ supportive services, 6) preventing or managing institutionalization, and 7) making lifestyle adjustments that support family care. Family caregiving facilitators included 1) faith and/ or faith communities, 2) having a healthcare background, and 3) support from local agencies and community programs.

**Conclusion:**

Though caregiver experiences align with resilience frameworks, systemic changes are necessary that engage wholistic care delivery models inclusive of multidisciplinary teams that center family as the unit of care. With appropriate multidisciplinary team engagement, families may be more readily connected to social and financial resources that support ongoing community living and quality of life.

